# The complete chloroplast genome sequence of *Quercus schottkyana,* and comparative analysis with related species

**DOI:** 10.1080/23802359.2021.1961630

**Published:** 2021-08-09

**Authors:** Tian-Tian Li, Chang-Sha Luo, Hong-Lin Mou, Xiao-Long Jiang, Gang-Biao Xu

**Affiliations:** The Laboratory of Forestry Genetics, Central South University of Forestry and Technology, Changsha, China

**Keywords:** Chloroplast genome phylogeny, *Quercus schottkyana*, Quercus section Cyclobalanopsis

## Abstract

In this study, we sequenced the complete chloroplast genome of *Quercus schottkyana.* The circular genome is 160,746 bp in size, featuring a typical quadripartite structure comprising one large single-copy region (LSC, 90,136 bp), one small single-copy region (SSC, 18,942 bp), and two copies of inverted repeat regions (IRs, 25,834 bp). The genome contains 131 genes, including 86 protein-coding genes, 37 transfer RNA genes, and 8 ribosomal RNA genes. The overall GC content is 36.90%. The maximum likelihood phylogenetic tree reconstructed using IQ-TREE indicated that *Q. schottkyana* has a closer relationship with *Quercus sichourensis* and *Quercus acuta*.

*Quercus* L. (Fagaceae) is the most diverse northern temperate tree genus comprising 400 tree and shrub species. Various *Quercus* spp. have been exploited for cultural and economic purposes for millennia (Gil-Pelegrin et al. 2017). According to the latest infrageneric classification, oaks including two subgenera, subgenus *Cerris* and *Quercus*. Subgenus *Cerris* contains three sections (*Cyclobalanopsis*, *Cerris* and *Ilex*) and is distributed in Eurasia, while subgenus *Quercus* is mainly distributed in America (Denk et al. 2017). *Quercus schottkyana* Rehder & E.H.Wilson 1916 belongs to *Quercus* section *Cyclobalanopsis.* The species has important ecological values, such as providing suitable habitats for other organisms, soil and water conservation, and maintaining the stability of regional climate. Meanwhile, the species is a precious wood species endemic to southwest China (Chen and Huang 1998). Therefore, understanding the plasmid genome of *Q. schottkyana* will be useful for future studies on the spatial genetic patterns of this species, as well as for the development and utilization of species resources. In this study, we sequenced, assembled, and annotated the complete chloroplast genome of *Q. schottkyana*, and constructed a phylogenetic tree to determine its phylogenetic position.

Fresh leaves were collected from a wild *Q. schottkyana* plant in the Yuanjiang County, Yunnan Province, China (23.66°N, 102.14°E, 1674 m). The voucher specimens were deposited in the Herbarium of Shanghai Chenshan Botanical Garden (CSH, http://csh.ibiodiversity.net/default.html, Contact Person: Bin-Jie GE, gebinjie123@163.com, under the voucher number DM19075). Total genomic DNA was extracted from silica-dried leaves using DNeasy plant tissue kit (TIANGEN Biotech Co., Ltd., Beijing) according to the manufacturer’s instructions. The total genomic DNA was preserved in the Laboratory of Forestry Genetics, Central South University of Forestry and Technology (Contact Person: Xiao-Long JIANG, xiaolongjiang1@gmail.com, under the accession number DM19075). Genome skimming sequencing was conducted using the Illumina NovaSeq 6000 platform. A total of 67,587,055 high-quality reads were retained after cleaning the raw reads using fastp v0.21.0 (Chen et al. 2018). To save time and computer resources, 5,000,000 reads were randomly selected from the clean reads and used for the *de novo* assembly with GetOrganelle v1.7.2beta (Jin et al. 2020). Chloroplast genome annotation was performed using Plastid Genome Annotator (PGA) (Qu et al. 2019).

The complete chloroplast genome of *Q. schottkyana* is a circular molecule of 160,746 bp in length. It exhibits a typical quadripartite structure, comprising a large single-copy (LSC) region (90,136 bp), a small single-copy (SSC) region (18,942 bp), and two inverted repeat regions (IR 25,834 bp). The overall GC content is 36.90%, whereas the corresponding values of the LSC, SSC, and IR regions are 34.77%, 30.55%, and 42.76%, respectively. A total of 131 genes were encoded: 86 are protein-coding genes, 37 are tRNA (transfer RNA) genes, and 8 are rRNA (ribosomal RNA) genes. The complete *Q. schottkyana* chloroplast genome sequence was submitted to GenBank with the accession number MW450872.

The chloroplast genome of *Q. schottkyana* and related species were used to infer the phylogenetic position of *Q. schottkyana*. A total of 25 chloroplast genome sequences related to *Q. schottkyana* were download from NCBI. Five species, *Trigonobalanus doichangensis* (A.Camus) Forman 1964*, Fagus crenata* Blume 1851, *Betula papyrifera* var. *cordifolia* (Regel) Regel 1865, *Platycarya strobilacea* Siebold & Zucc. 1843 and *Juglans mandshurica* Maxim. 1856 were selected as outgroups. MAFFT v7.4.75 was used to align the chloroplast genome sequences (Rozewicki et al. 2019). A maximum likelihood (ML) tree of *Q. schottkyana* was reconstructed using IQ-TREE v1.6.12 (Chernomor et al. 2016). Node support was assessed by 1000 fast bootstrap replicates. The result indicated that *Q. schottkyana* is more closely related to *Q. sichourensis* and *Q. acuta* with 98% bootstrap support (Figure 1).

**Figure 1.**
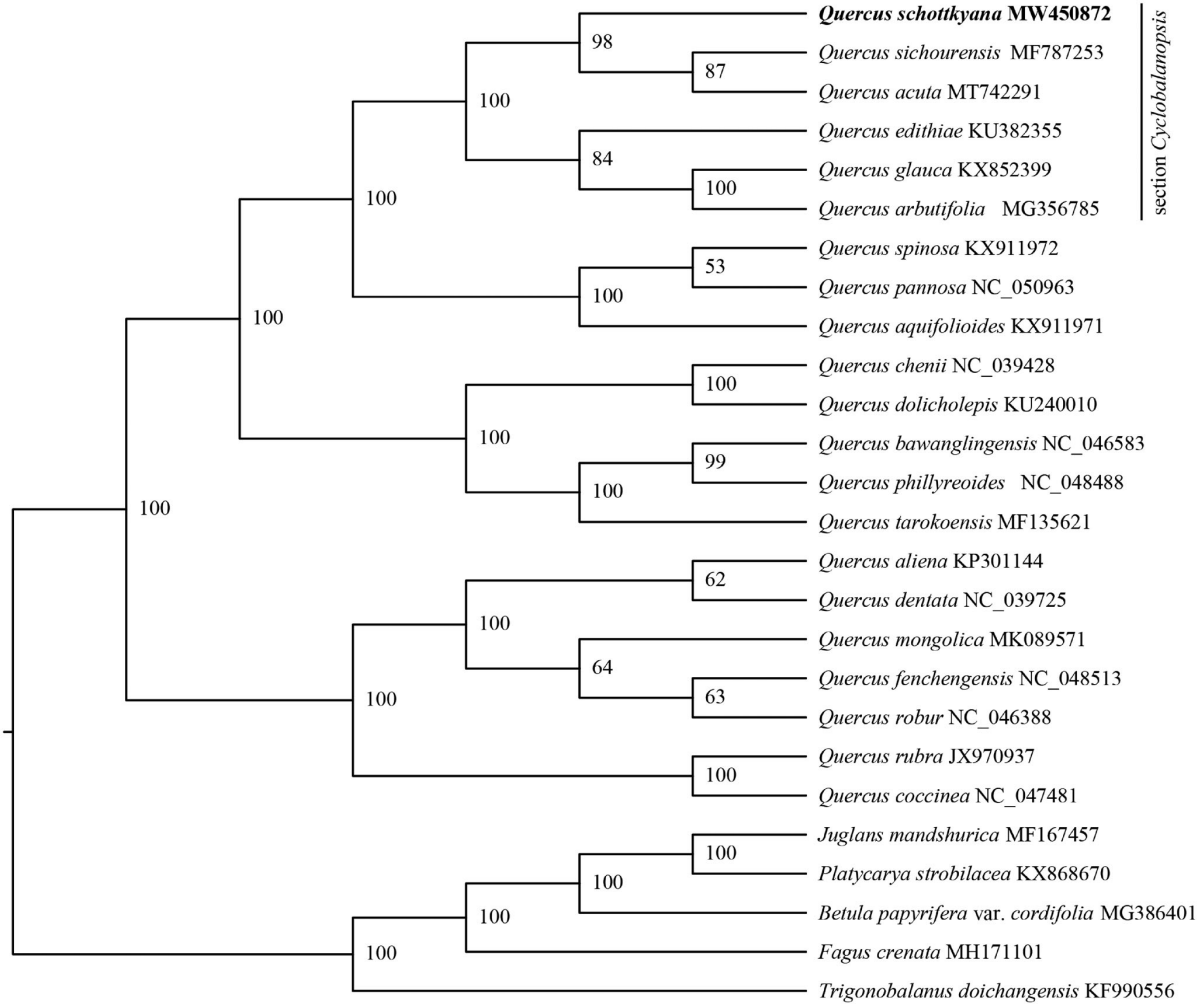
The maximum likelihood (ML) phylogenetic tree of *Quercus.schottkyana* and 25 relative species were reconstructed by IQ-TREE based on complete chloroplast genome sequences. The bootstrap support value is labeled for each node.

## Data Availability

The complete chloroplast genome sequence of *Quercus schottkyana* is deposited in the GenBank database under the accession number MW450872 (https://www.ncbi.nlm.nih.gov/nuccore/MW450872). The associated BioProject, SRA, and Bio-Sample numbers are PRJNA725598, SRR14338617, and SAMN17905928, respectively.
